# *In vivo* characterization of sAC null sperm

**DOI:** 10.3389/fcell.2023.1134051

**Published:** 2023-04-21

**Authors:** Carla Ritagliati, Sylvia Ayoub, Melanie Balbach, Jochen Buck, Lonny R. Levin

**Affiliations:** Department of Pharmacology, Weill Cornell Medicine, New York, NY, United States

**Keywords:** *in vivo* migration, motility, soluble adenylyl cyclase, hyperactivation, acrosome reaction

## Abstract

Targeted disruption of the soluble adenylyl cyclase (ADCY10; sAC) gene results in male-specific sterility without affecting spermatogenesis, mating behavior, or spermatozoa morphology and count; however, it dramatically impairs sperm motility and prevents capacitation. These phenotypes were identified in sperm from sAC null mice surgically extracted from the epididymis and studied *in vitro*. Epididymal sperm are dormant, and never exposed to physiological activators in semen or the female reproductive tract. To study sAC null sperm under conditions which more closely resemble natural fertilization, we explored phenotypes of ejaculated sAC null sperm *in vivo* post-coitally as well as *ex vivo*, collected from the female reproductive tract. *Ex vivo* ejaculated sAC null sperm behaved similarly to epididymal sAC null sperm, except with respect to the physiologically induced acrosome reaction. These studies suggest there is a sAC-independent regulation of acrosome responsiveness induced upon ejaculation or exposure to factors in the female reproductive tract. We also studied the behavior of sAC null sperm *in vivo* post-coitally by taking advantage of transgenes with fluorescently labelled sperm. Transgenes expressing GFP in the acrosome and DsRed2 in the mitochondria located in the midpiece of sperm (DsRed2/Acr3-EGFP) allow visualization of sperm migration through the female reproductive tract after copulation. As previously reported, sperm from wild type (WT) double transgenic mice migrated from the uterus through the uterotubular junction (UTJ) into the oviduct within an hour post-copulation. In contrast, sperm from sAC null double transgenic mice were only found in the uterus. There were no sAC null sperm in the oviduct, even 8 h after copulation. These results demonstrate that sAC KO males are infertile because their sperm do not migrate to the fertilization site.

## 1 Introduction

Morphologically mature sperm are stored, in a dormant state, in the cauda epididymis which is characterized by an extremely low (≤5 mM) bicarbonate (HCO_3_^−^) concentration (Levine and Kelly, 1978). Upon ejaculation, sperm mix with seminal plasma, which contains 25 mM bicarbonate along with various nutrients that initiate and support sperm motility. Ejaculated motile sperm are still not capable of fertilizing an oocyte. Mammalian sperm acquire fertilization competence in the female genital tract, in a process known as capacitation, which involves molecular, cellular, and physiological changes. During this process sperm increase their flagellar beat frequency; switch their motility pattern to a fast whip-like asymmetrical movement, known as hyperactivation; and gain acrosomal responsiveness to a physiological stimulus.

Studying sperm physiology is greatly enhanced by using mouse genetics to study phenotypes of sperm devoid of key proteins. For most studies, sperm are surgically isolated from the epididymis, and the process of capacitation is defined by conditions which are sufficient to support sperm functions essential for *in vitro* fertilization. While such studies have revealed molecular hallmarks of capacitation, they may miss physiologically relevant processes that sperm undergo prior to fertilizing the oocyte in the oviduct *in vivo.* For example, migration through the female reproductive tract is a key element of natural fertilization which is not mimicked in the test tube.

After being deposited in the vagina during coitus, mammalian sperm must travel through the female’s reproductive tract to reach the site of fertilization, the ampullary region of the uterine tube. To reach and penetrate the oocyte, sperm migration along the female tract is thought to depend upon contractions of the myometrium (Lyons et al., 1991; Kunz et al., 1996; Ishikawa et al., 2016), interactions between sperm and the female tract (Yamaguchi et al., 2009; Fujihara et al., 2013; Okabe, 2015) and sperm motility (Gaddum-Rosse, 1981). Among the semen parameters motility is considered to be a strong predictor of male fertility potential. Sperm dysfunction has consistently been identified as a common cause of infertility, with asthenozoospermia (the medical term for reduced sperm motility), among the predominant contributing factors for male infertility (Dcunha et al., 2022).

The complex signaling cascade leading to initiation of motility and capacitation is stimulated upon ejaculation when cauda epididymal sperm encounter seminal plasma, where elevated HCO_3_^−^ activates soluble adenylyl cyclase (sAC or ADCY10) (Visconti et al., 1995; 2011; Chen et al., 2000). sAC activity is directly stimulated by bicarbonate (Chen et al., 2000; Kleinboelting et al., 2014), which induces an allosteric change facilitating catalysis (Kleinboelting et al., 2014). In the absence of sAC, mammalian sperm are unable to fertilize, and sAC knock-out (KO) mice exhibit male-specific sterility. Targeted disruption of the sAC gene or pharmacological inhibition of sAC does not affect spermatogenesis, mating behavior, nor spermatozoa morphology and count; however, it dramatically impairs sperm motility, prevents capacitation, and results in sperm which cannot fertilize zona-intact oocytes (Esposito et al., 2004; Xie et al., 2006; Marquez and Suarez, 2008; Ramos-Espiritu et al., 2016; Balbach et al., 2021; 2023). These deficits were identified by studying epididymal sAC null sperm; thus, until now, mouse sAC null sperm were only studied in the context of *in vitro* capacitating conditions.

The role of sAC in male fertility was also genetically and pharmacologically confirmed in humans (Akbari et al., 2019; Balbach et al., 2021; 2023). Two infertile male patients were found to be homozygous for a frameshift mutation in the exonic region of ADCY10, leading to premature termination and interruption of the catalytic domains. Sperm from these patients are immotile, similar to sperm from sAC KO mice, and their motility defect could be rescued with cell-permeable cAMP analogs, consistent with the loss of sAC disrupting motility and causing infertility (Akbari et al., 2019). Interestingly, unlike mouse sAC null sperm which were studied only after exposure to bicarbonate in the capacitating media *in vitro,* human sAC null sperm were studied following ejaculation, after the sperm were already exposed to the bicarbonate and nutrients present in seminal plasma.

Two advances in mouse physiology facilitate studying sperm in more physiological contexts. Post-ejaculated mouse sperm can be studied *ex vivo* and *in vivo.* Recovering ejaculated mouse sperm from the female reproductive tract post-coitus affords the opportunity to study mouse sperm *ex vivo* after exposure to seminal plasma as well as factors derived from the female reproductive tract. *In vivo*, mouse sperm migration through the female reproductive tract can be visualized post-coitally with the aid of fluorescent markers. Using these methods to more accurately reflect natural fertilization, we characterized the *in vivo* phenotypes of sAC null sperm.

## 2 Materials and methods

### 2.1 Animals

The transgenic C1 sAC null mouse line, in which exons 2–4 are deleted, was generated at Lexicon Genetics (The Woodlands, TX). The transgenic mouse line whose sperm express green fluorescent protein (GFP) in their acrosome and red fluorescent protein (RFP) in their mitochondria (B6D2F1-Tg (CAG/Su9-DsRed2, Acr3-EGFP) RBGS002Osb) (Hasuwa et al., 2010) is from M. Okabe (Osaka U, Japan) via the RIKEN Institute. Animal experiments were approved by Weill Cornell Medicine’s Institutional Animal Care and Use Committee (IACUC).

### 2.2 Reagents

3-Isobutyl-1-methylxanthine (IBMX), bovine serum albumin (BSA), NaHCO_3_ and Progesterone were purchased from Sigma-Aldrich (St. Louis, MO, United States). 8-Bromo-cAMP (sodium salt) and cAMP Elisa kit from Cayman Chemical (Ann Arbor, MI, United States). *β*-mercaptoethanol from Thermo Fisher Scientific (Waltham, MA, United States). PBS buffer was purchased from Corning (Radnor, PA, United States). Rabbit monoclonal anti-phospho-PKA substrates (anti-pPKA substrates) (clone 100G7E) and Horseradish peroxidase-conjugated anti-mouse and anti-rabbit IgG, were purchased from Cell Signaling Technology (Danvers, MA). Mouse anti-phospho Tyrosine (anti-pTyr) (clone 4G10) was from EMD Millipore.

### 2.3 Generation of the double fluorescent transgenic sAC KO line

Crossing sAC KO females with Acr-EGFP/Su9-DsRed2 males generated Acr-EGFP/Su9-DsRed2/sAC^−/−^ mice. The presence of the transgenes expressing fluorescent tags as well as disruption of sAC gene in the germline were confirmed by PCR analysis.

### 2.4 Sperm preparation

Mouse sperm were isolated by incision of the cauda epididymis of male mice or from the female genital tract after copulation, followed by a swim-out in 500 μL Toyoda Yokoyama Hoshi (TYH) medium (in mM: 119.3 NaCl, 4.7 KCl, 1.7 CaCl_2_, 1.2 KH_2_PO_4_, 1.2 MgSO_4_, 5.6 glucose, 0.5 pyruvate, 20 HEPES, pH 7.4 adjusted with NaOH), prewarmed at 37°C and counted using a hematocytometer. For capacitation, sperm were incubated in TYH containing 5 mg/mL BSA and 20 mM NaHCO_3_ or 0.5 mM 8Br-cAMP and 0.5 μM IBMX at 37°C.

### 2.5 Sperm cAMP quantification

Aliquots of 3−4 x 10^6^ WT or sAC^−/−^ double transgenic sperm were incubated for 15 min in the presence of 0.5 μM IBMX in non-capacitating (TYH-) or capacitating (TYH + HCO_3_/BSA) buffer. Sperm were sedimented by centrifugation at 2000 x g for 3 min and lysed in 50–100 μL of 0.1 M HCl for 10 min. Sperm lysates were centrifuged at 2000 x g for 3 min and the cAMP in the supernatant was acetylated and quantified using the Direct cAMP ELISA Kit (Cayman) according to the manufacturer’s instructions.

### 2.6 Western blot analysis

Aliquots of 1−2 x 10^6^ WT or sAC^−/−^ double transgenic sperm were incubated for 60 min in TYH buffer in the absence or presence of 5 mg/mL BSA and 20 mM NaHCO_3_ or 0.5 mM 8Br-cAMP and 0.5 μM IBMX. Sperm were collected by centrifugation for 3 min at 2000 x g, washed twice with 1 mL TBS, and resuspended with Laemmli sample buffer without *β*-mercaptoethanol, vortexed for 10 s and boiled for 3 min. After centrifugation, 5% *β*-mercaptoethanol was added to the supernatants and boiled again for 5 min. Protein extracts equivalent to 0.5−1 x 10^6^ sperm/lane were subjected to SDS-PAGE and electrotransferred to PVDF membranes at 250 mA for 90 min. Membranes were blocked with 3% BSA in TBS containing 0.1% Tween-20 (T-TBS). Antibodies were diluted in T-TBS as follows: 1/5,000 for anti-pTyr and 1/3,000 for anti-pPKA substrates. Secondary antibodies were diluted 1/10,000 in T-TBS and the membranes analyzed using an enhanced chemiluminescence detection kit (SuperSignal West Femto Maximun Sensitivity Substrate, Thermo Scientific) according to manufacturer’s instructions.

### 2.7 Sperm motility analysis

Sperm suspensions were loaded on a 100-μm deep Leja slides and placed on a microscope stage at 37°C. Sperm movements were examined using an IVOSII Hamilton Thorne. Parameters used were as follows: 45 frames acquired, frame rate of 60 Hz, and cell size of 25–200 μm^2^. At least 5 microscopy fields and a minimum of 500 sperm were analyzed in each experiment. The following parameters were measured: mean path velocity (VAP, μm/sec), curvilinear velocity (VCL, μm/sec), straight-line velocity (VSL, μm/sec), linearity (LIN, %), amplitude of lateral head displacement (ALH, μm), beat cross frequency (BCF, Hz) and straightness (STR, %). Sperm were considered hyperactivated when presenting VCL ≥271 μm/s, LIN <50%, and ALH ≥3.5 μm and progressive when presenting VAP ≥50 μm/s and STR >80%.

### 2.8 Acrosomal status assay

After incubating WT and sAC^−/−^ sperm under non-capacitating or capacitating (+HCO_3_^−^/BSA or +8Br-cAMP/IBMX) conditions for 60 min, sperm were incubated for an additional 30 min in the absence or presence of 25 μM progesterone (Pg). Sperm were sedimented by centrifugation at 1,000 x g for 5 min, washed twice with PBS, and seeded on 8-well glass slides. After adhesion, sperm were incubated with PBS containing DAPI for 10 min. Before mounting, samples were washed with PBS (four times for 5 min each time). Epifluorescence microscopy was used to assess acrosomal status, distinguished by the presence or absence of green fluorescence of intact acrosomes. At least 300 sperm were analyzed in each condition.

### 2.9 *In vivo* sperm migration assay

Single-caged double transgenic DsRed2/Acr3-EGFP WT or sAC^−/−^ males were combined with WT females in estrous and vaginal plug formation was checked every 30 min. Once plug formation was confirmed, the male was separated from the female. The uteri and oviducts were excised various times after coitus (1.5, 4, and 8 h), transferred to slides, and examined for the presence of fluorescent sperm expressing mitochondrial DsRed2 and/or acrosomal EGFP markers in the female reproductive tract (IX73 Olympus). The experiments were repeated at least 3 times with 3 different males.

## 3 Results

### 3.1 Fluorescently tagged double transgenic sAC KO sperm do not display molecular hallmarks associated with capacitation

To be able to study sAC null sperm *in vivo,* we crossed transgenes which label the acrosome and mitochondria (located in the midpiece) of spermatozoa with green and red fluorescent proteins, respectively (DsRed2/Acr3-EGFP) (Hasuwa et al., 2010), into sAC^−/−^ (KO) mice (Esposito et al., 2004; Hess et al., 2005) as well as into our isogenic C57Bl/6 strain. The resultant double transgenic sAC KO mouse line exhibited no overt developmental abnormalities. As previously reported, sAC KO males were infertile despite producing epididymal spermatozoa with the same count and morphology as wild type (WT) males (Supplementary Figures S1A, B) and showing normal mating behavior with successful ejaculation and vaginal plug formation (Supplementary Figure S1C).

**FIGURE 1:**
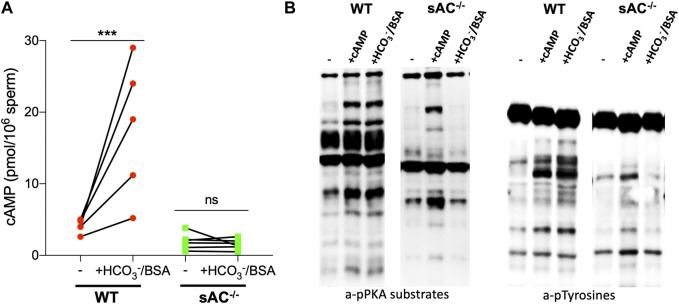
**(A)** Intracellular cAMP levels in WT (red circles) and sAC^−/−^ (green squares) sperm after incubation for 15 min in the presence of 0.5 μM IBMX in non-capacitating (−) or capacitating (+HCO_3_^−^/BSA) media. Individual replicates indicated by symbols, N = 5. Ratio paired Student’s t test was performed between indicated conditions: ***p < 0.001, ns (non-significant). **(B)** Western blot analysis using anti-pPKA substrates and anti-pTyrosines antibodies. Sperm were incubated for 60 min in the absence (−) or presence of 0.5 mM 8Br-cAMP and 0.5 μM IBMX (+cAMP) or 20 mM HCO_3_^−^ and 5 mg/mL BSA (+HCO_3_^−^/BSA).

Also similar to spermatozoa from sAC KO mice, epididymal spermatozoa from DsRed2/Acr3-EGFP/sAC KO males did not display molecular hallmarks normally associated with capacitation when incubated in the presence of HCO_3_^−^: they did not increase cAMP levels and they did not produce the prototypical pattern of phosphorylation of PKA substrates and tyrosines. As expected, addition of membrane-permeable cAMP, the product of sAC, rescued the phosphorylation of PKA substrates and tyrosines (Figure 1), confirming that any defects observed in sperm from double transgenic sAC KO mice come from the absence of sAC. These results validate that the double transgenic, fluorescently tagged sAC KO line phenotypically reflect previously characterized sAC KO mice (Esposito et al., 2004; Hess et al., 2005; Xie et al., 2006).

### 3.2 Both epididymal and ejaculated double transgenic sAC KO sperm are immotile

**FIGURE 2:**
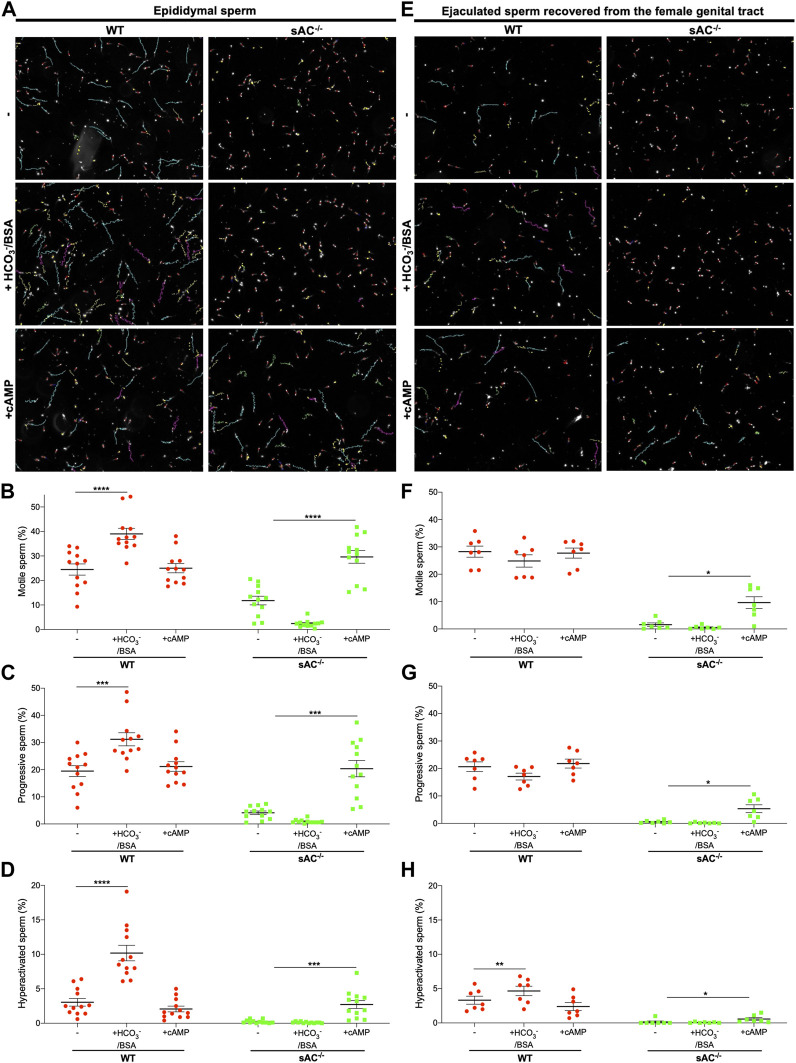
Epididymal and ejaculated WT (red circles) and sAC^−/−^ (green squares) sperm incubated in the absence (−) or presence of 20 mM HCO_3_^−^ and 5 mg/mL BSA (+HCO_3_^−^/BSA) or 0.5 mM 8Br-cAMP and 0.5 μM IBMX (+cAMP) for 20 min **(A,E)** Representative images with motility tracks of the CASA obtained with IVOSII Hamilton Thorne. Track´s color code: motile (green), progressive (turquoise), hyperactivated (pink), static (red), not counted (yellow). Bar graphs show percent total motility **(B,F)**, progressive motility **(C,G)** and hyperactivation **(D,H)** with the individual values for each experiment indicated. Data shown as mean ± SEM, representative of at least 7 independent experiments. More than 500 sperm in at least 5 fields were analyzed. Hyperactivated sperm are defined by: VCL ≥271 μm/s, LIN <50%, and ALH ≥5 μm.

Sperm surgically extracted from the epididymis are isolated prior to ejaculation so they have never been exposed to the HCO_3_^−^ in seminal plasma. Like other WT epididymal sperm, epididymal sperm from double transgenic WT males are motile, and the fraction of sperm exhibiting hyperactivated motility increases in the presence of HCO_3_^−^ (Figure 2; Supplementary Movies S1, S2). To compare epididymal sperm with ejaculated mouse sperm, we mated double transgenic males with WT females and recovered sperm from the uterus post-copulation. As expected, ejaculated WT sperm were motile, but remarkably they did not display the bicarbonate-induced increases in total and progressive motility seen in epididymal sperm. In addition, they showed only a slight increase in hyperactivation (Figure 2; Supplementary Movies S1, S2). Presumably ejaculated sperm are less sensitive to bicarbonate because they have already been exposed to bicarbonate-dependent stimulation upon mixing with seminal plasma and fluids in the female tract.

We used the same methods to isolate and characterize ejaculated sAC null sperm. When we mated sAC^−/−^ double transgenic males with WT females, we observed *bona fide* plugs and plumped uteri after copulation (Supplementary Figure S2), and we successfully obtained sAC null sperm from uteri of receptive females. We recovered the same amount of ejaculated sperm from the female tract after mating with WT and sAC KO males (Supplementary Figure S2), confirming that sAC ablation does not affect mating or ejaculatory behaviors. In agreement with previous reports for epididymal sAC KO sperm (Esposito et al., 2004; Hess et al., 2005; Marquez and Suarez, 2008), both epididymal and ejaculated double transgenic sAC^−/−^ sperm were immotile, showing only vibratory movements (Figure 2; Supplementary Movies S1, S2). Motility was restored by addition of a cell-permeable cAMP analog, proving the motility defect is due to the absence of sAC. Thus, double-transgenic sAC KO sperm are immotile even after ejaculation.

### 3.3 Ejaculated sAC KO sperm display bicarbonate-independent acrosomal responsiveness to progesterone

**FIGURE 3:**
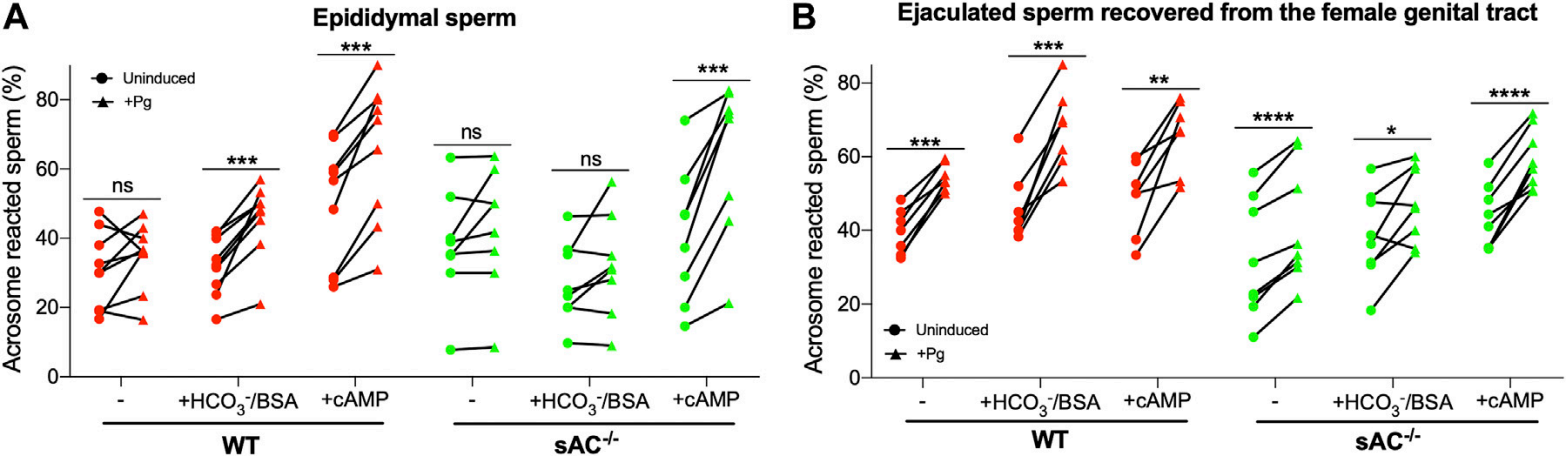
Progesterone-induced AR of epididymal **(A)** and ejaculated **(B)** WT (red symbols) and sAC^−/−^ (green symbols) sperm incubated in the absence (−) or presence of 20 mM HCO_3_^−^ and 5 mg/mL BSA (+HCO_3_^−^/BSA) or 0.5 mM 8Br-cAMP and 0.5 μM IBMX (+cAMP) for 60 min, 25 μM Progesterone (+Pg) or DMSO (Uninduced) were added, and then further incubated for 30 min. Data shown as mean ± SEM, representative of at least 7 independent experiments. Paired Student’s t test was performed between indicated conditions: *p < 0.05, **p < 0.005, ***p < 0.001, ns (non-significant).

Another hallmark of capacitation is the ability to undergo a physiologically induced acrosome reaction (AR). WT epididymal sperm exhibited progesterone (Pg) induced AR following incubation with bicarbonate or after addition of exogenous membrane permeable cAMP (Figure 3A). In contrast, with epididymal sAC KO sperm, we only observed the Pg-induced AR in the presence of exogenous cAMP. These data demonstrate that there is a cAMP-dependent component to enable acrosomal responsiveness, which is mediated via sAC following bicarbonate stimulation. In ejaculated WT sperm, which were already exposed to bicarbonate in seminal plasma and fluids in the female reproductive tract, acrosomal responsiveness to Pg was observed in non-capacitating media (Figure 3B). Addition of bicarbonate enhanced the Pg-induced increase in AR (Supplementary Figure S3). Surprisingly, ejaculated sAC null sperm displayed a degree of Pg responsiveness in both non-capacitating and capacitating media (Figure 3). Thus, there also appears to be a sAC-independent mechanism regulating AR associated with ejaculation or residence in the female reproductive tract. As expected, epididymal and ejaculated sAC KO sperm lost any bicarbonate-induced increase in Pg-stimulated AR (Supplementary Figure S3), but in both cases, Pg responsiveness was increased by cell-permeable cAMP, corroborating the idea that there are both cAMP-dependent and cAMP-independent mechanisms for increasing the response to Pg.

### 3.4 sAC KO sperm do not progress into the oviduct

**FIGURE 4:**
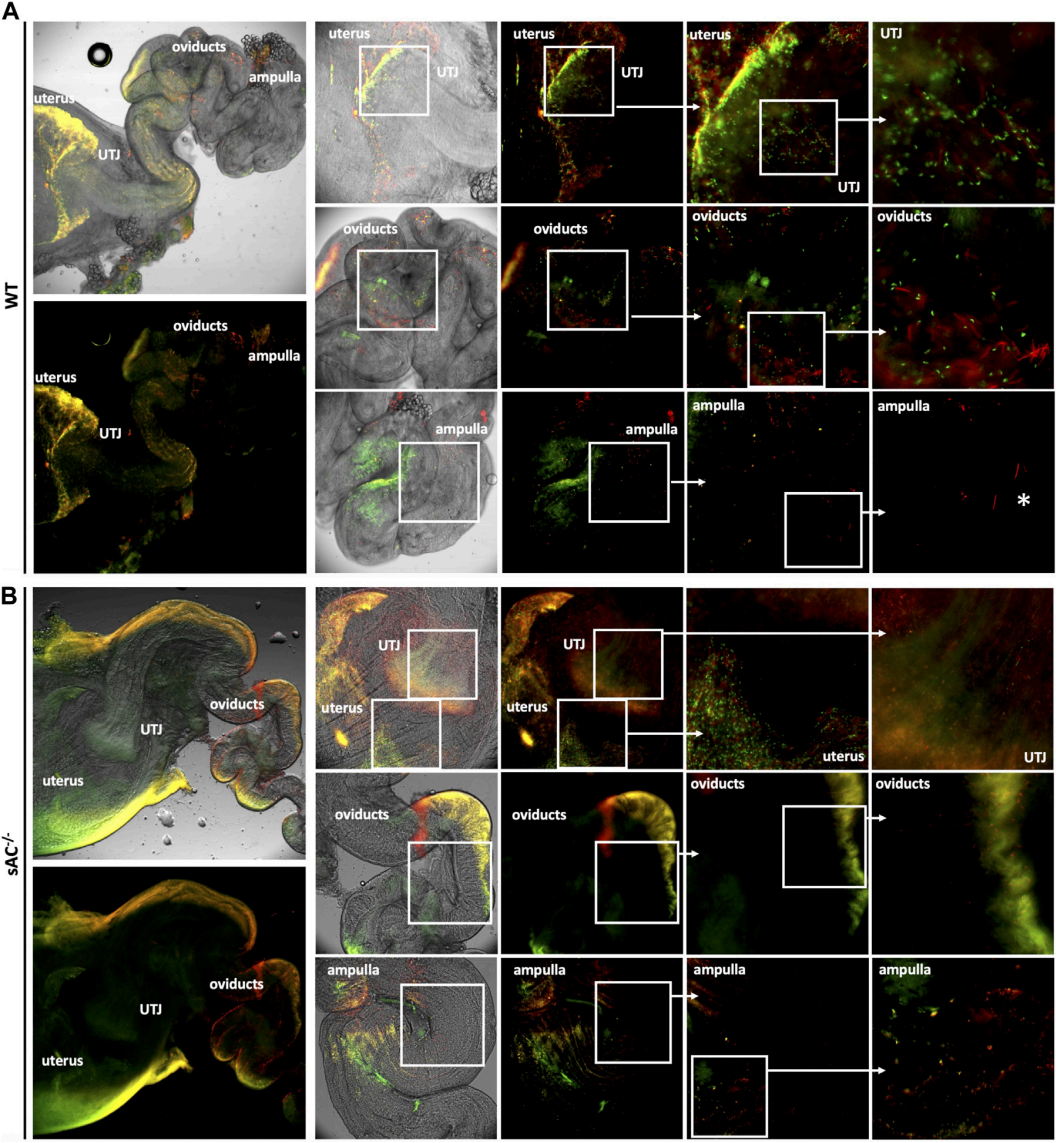
Migration of DsRed2/Acr3-EGFP/WT **(A)** and sAC^−/−^ **(B)** sperm in the female genital tract 4 h post-copulation. Males were caged with WT females in estrous and after vaginal plug detection, they were separated. Four hours post-copulation, the female genital tracts were dissected and mounted for fluorescence microscopy analysis using an IX73 Olympus microscope. The indicated squared regions were digitally magnified and shown to the right. White asterisks depict acrosome reacted WT sperm in the ampulla. Images representative of at least 3 independent experiments.

Finally, to explore the *in vivo* roll of sAC, we studied sperm migration through the female reproductive tract following matings between WT or sAC KO double transgenic males with receptive WT females. Sperm ejaculated from WT mice migrated from the uterus through the uterotubular junction (UTJ) into the oviduct within an hour post-copulation, and by 4 h sperm were observed all the way from the oviduct to the ampulla. In contrast, while sperm ejaculated from sAC^−/−^ double transgenic mice were present inside the uterus, few were infrequently observed in the UTJ, and none successfully progressed into the oviduct even 8 h after copulation (Figure 4; Supplementary Figures S4, S5).

## 4 Discussion

sAC null male mice and men are infertile (Esposito et al., 2004; Hess et al., 2005; Akbari et al., 2019)*.* Previously, the specific defects in sAC null mouse sperm were studied in sperm isolated from the epididymis. Epididymal sperm are dormant, the signaling cascades which initiate motility and capacitation are activated by factors in seminal plasma and/or the female reproductive tract. Epididymal sperm genetically or pharmacologically devoid of sAC activity are immotile, do not capacitate, and do not fertilize oocytes in mouse IVF experiments (Esposito et al., 2004; Hess et al., 2005; Xie et al., 2006; Marquez and Suarez, 2008; Ramos-Espiritu et al., 2016; Balbach et al., 2021; 2023). We now explored phenotypes of sAC null sperm in states which better reflect natural fertilization. sAC null sperm collected post-coitally from the female reproductive tract revealed the existence of a sAC-independent, acrosome responsiveness in ejaculated sperm, and using transgenic mice expressing GFP and DsRed we demonstrate that sAC null sperm are unable to penetrate beyond the UTJ to reach the fertilization site.

As expected, sAC KO mouse sperm do not respond to bicarbonate in capacitating media. Neither epididymal nor ejaculated sAC KO sperm altered motility or acrosomal responsiveness in media containing bicarbonate. This observation reinforces the idea that sAC mediates the bicarbonate-induced changes during capacitation (Visconti et al., 1999; 2011; Ferreira et al., 2021). However, unlike epididymal sAC null sperm, ejaculated sAC null sperm recovered from the female genital tract can undergo a low level of progesterone-induced acrosome reaction. Thus, there appears to be a sAC-independent mechanism caused by ejaculation and/or residence in the female genital tract.

A major difference between mouse and human reproduction is the anatomy of the female reproductive tract. In humans, females possess a cervical restriction, and it is speculated that the sperm’s progressive motility allows them to pass through the cervix to escape the inhospitable environment of the vagina. In contrast, mouse females lack a cervical restriction so ejaculated sperm deposited in the vagina are rapidly swept unimpeded into the uterus. Sperm accumulate at the UTJ, which can be considered a major selective barrier to mouse sperm migration through the female reproductive tract drastically reducing the number of sperm entering the oviduct (Suarez and Pacey, 2006; Mahé et al., 2021). Because immotile sAC null sperm fail to cross beyond the UTJ, we conclude that sperm must at least have progressive motility to pass through the UTJ.

There are three processes which appear to contribute to sperm’s ability to travel through the female reproductive tract to reach the fertilization site: interactions between sperm and the female tract (Yamaguchi et al., 2009; Fujihara et al., 2013; Okabe, 2015), contractions of the myometrium and oviduct (myosalpinx) (Lyons et al., 1991; Kunz et al., 1996; Ishikawa et al., 2016), and sperm motility (Gaddum-Rosse, 1981). A number of distinct KO mouse lines with defects in binding to the oocyte’s zona pellucida fail to enter the UTJ (Okabe, 2015). These lines display no detectable abnormalities in motility or morphology, implying that their migration defect is due to a lack of specific interactions between sperm and the epithelium of the female tract (Muro et al., 2016). These interactions seem to be essential for successful sperm entry into and passing through the UTJ. Once across the UTJ, myosalpinx contractions help the transport of sperm from the uterus to the isthmus and the middle region of the oviduct (Ishikawa et al., 2016).

Completely immotile rat sperm failed to cross the UTJ (Gaddum-Rosse, 1981); thus, it was proposed that most spermatozoa require self-propulsion to get through the UTJ into the oviduct. However, this study relied upon killing sperm to render them immotile. CatSper1-null sperm, which are motile but do not hyperactivate, can migrate through the UTJ and reach the ampulla, though less effectively (Chung et al., 2014). A number of mouse strains with KOs of sperm-specific metabolic enzymes (phosphoglycerate Kinase 2, glyceraldehyde 3-phosphate dehydrogenase, and lactate dehydrogenase C) display sperm motility defects which correlate to male-specific infertility (Miki et al., 2004; Odet et al., 2008; Danshina et al., 2010), but *in vivo* migration has not been studied. We predict that migration of sperm from these strains will be impaired due to their motility defect. In our study, sAC null sperm, which are alive but immotile, are unable to migrate through the UTJ to the fertilization site.

While the UTJ represents the major barrier to sperm migration in rodents, the cervical restriction represents the major barrier to sperm migration in humans. Our demonstration that sAC KO male mice are infertile because their sperm do not penetrate beyond the UTJ suggests that human’s sAC KO men are infertile because their sperm will not cross the cervix to enter the uterus to be able to ever reach the oviduct and the fertilization site. Because the vagina reacidifies following intercourse, in the absence of sAC, human sperm are not likely to survive long after sex.

## Data Availability

The raw data supporting the conclusion of this article will be made available by the authors, without undue reservation.
