# Supplementary material for: In vivo characterization of sAC null sperm
Source: Front Cell Dev Biol. 2023 Apr 21;11:1134051. doi: 10.3389/fcell.2023.1134051 (PMC10160483; doi:10.3389/fcell.2023.1134051)
Supplement: Supplementary file 3 [file DataSheet1.DOCX]

**Supplementary Material**

**In vivo characterization of sAC null sperm.**

Ritagliati, Carla^1^, Ayoub, Sylvia^1^, Balbach, Melanie^1^, Buck, Jochen^1^, Levin, Lonny R^1,*^.

**^*^ Correspondence:** Lonny R. Levin. [llevin@med.cornell.edu](mailto:llevin@med.cornell.edu)


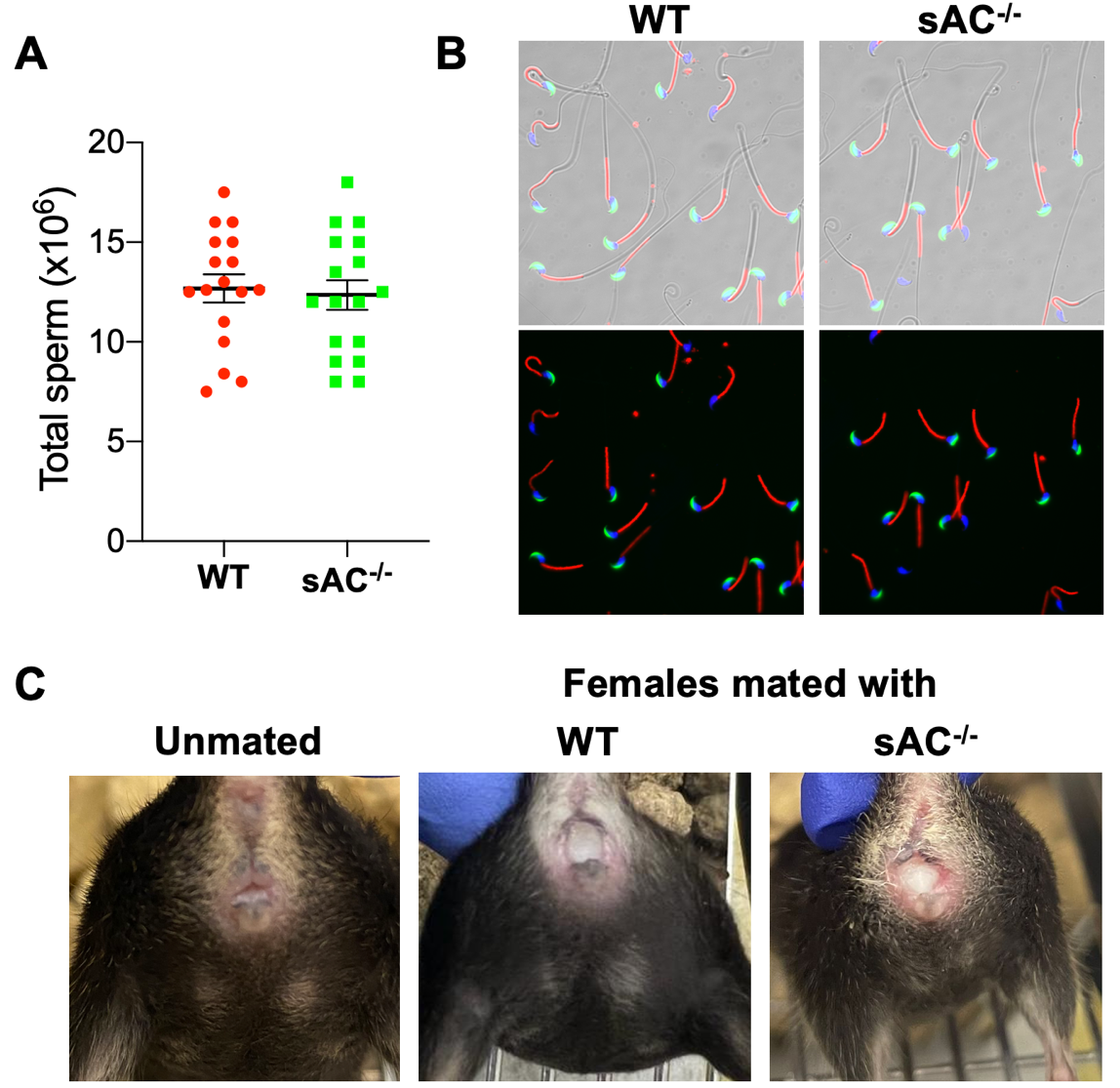


**Figure S1. (A)** Total number of epididymal sperm recovered from WT (red circles) and sAC^-/-^ (green squares) males. Data represented as mean ± SEM (n = 17 with individual replicates indicated by symbols). (**B)** Fluorescent images of DsRed2/Acr3-EGFP WT and sAC^-/-^ sperm. The DNA was stained with DAPI (blue). The acrosomes are shown in green and the mitochondria in red. (**C)** Representative images of vaginal plugs observed after mating WT females with DsRed2/Acr3-EGFP WT and sAC^-/-^ males. As a control an image of an unmated female was included.


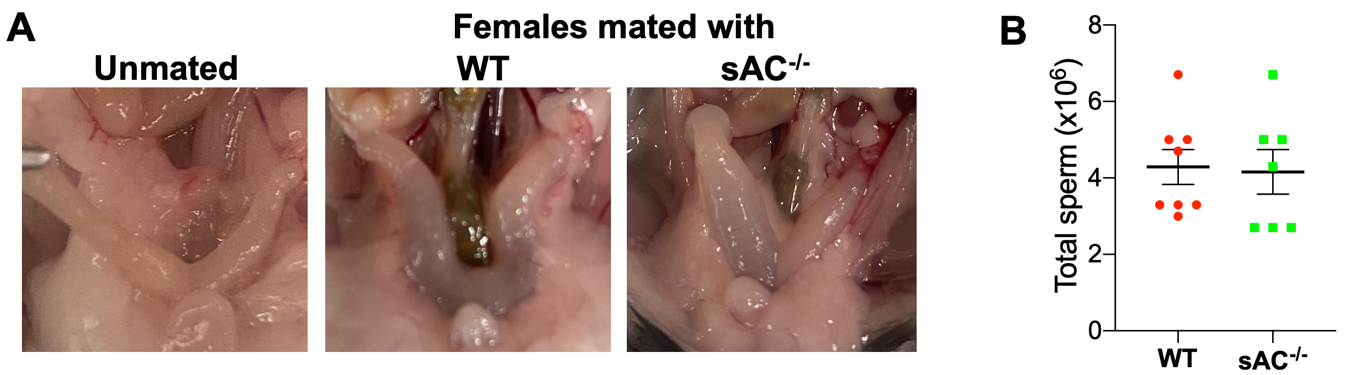


**Figure S2. A)** Representative images of the uteri of WT females after mating with DsRed2/Acr3-EGFP WT and sAC^-/-^  males. As a control an image of an unmated female was included. **B)** Total number of ejaculated sperm recovered from the female tract from WT (red circles) and sAC^-/-^ (green squares) males. Data represented as mean ± SEM (n ≥ 7 with individual replicates indicated by symbols).


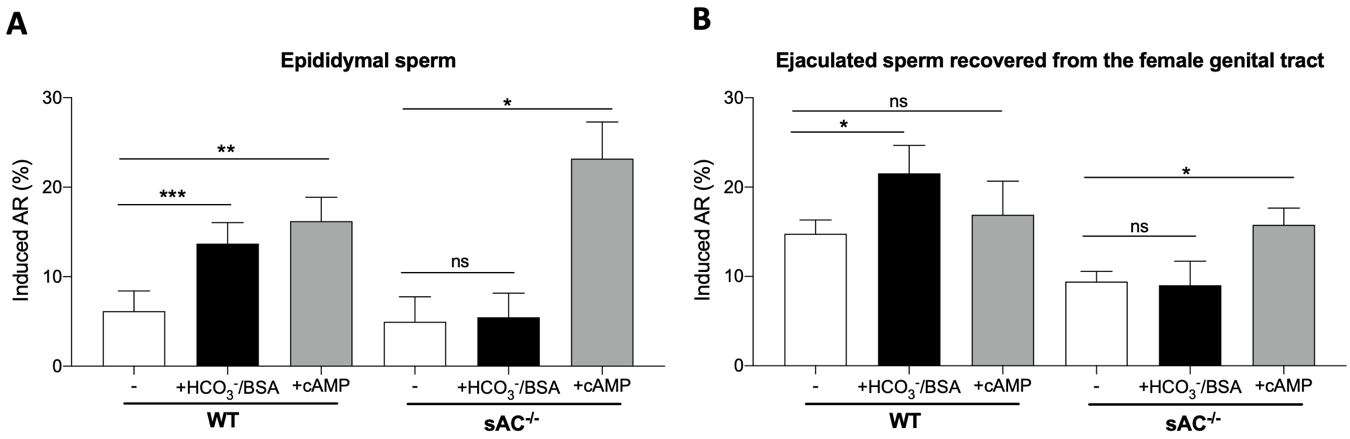


**Figure S3.** Induced acrosome reaction (AR) in epididymal (A) and ejaculated (B) WT and sAC^-/-^ sperm. Values represent the difference between Pg induced and basal (uninduced) AR calculated from the data in Figure 3.


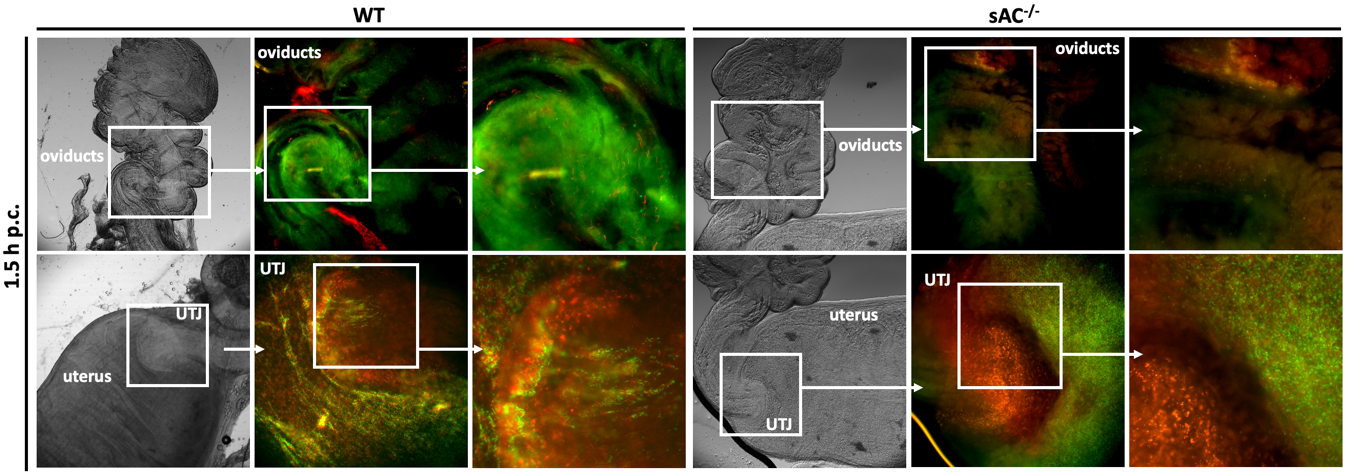


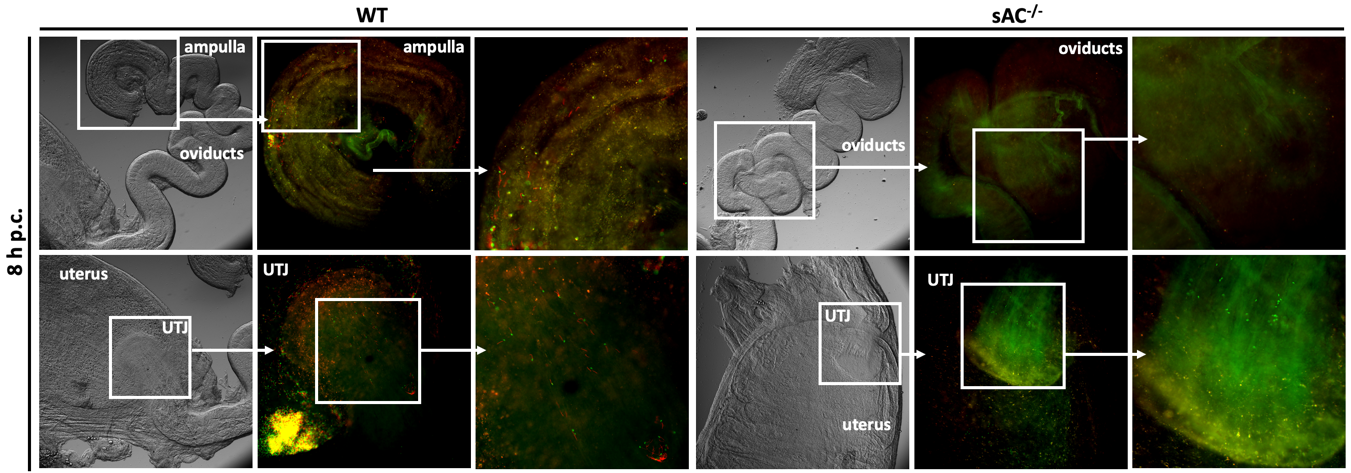


**Figure S4.** DsRed2/Acr3-EGFP/ WT and sAC^-/-^ males were paired with WT females in estrous and after vaginal plug detection, mice were separated. At 1.5 and 8 hours post-copulation (p.c.), the female genital tracts were dissected and mounted for fluorescence microscopy analysis using an IX73 Olympus microscope. The indicated squared regions were digitally magnified and shown to the right. Images representative of at least 3 independent experiments are shown.


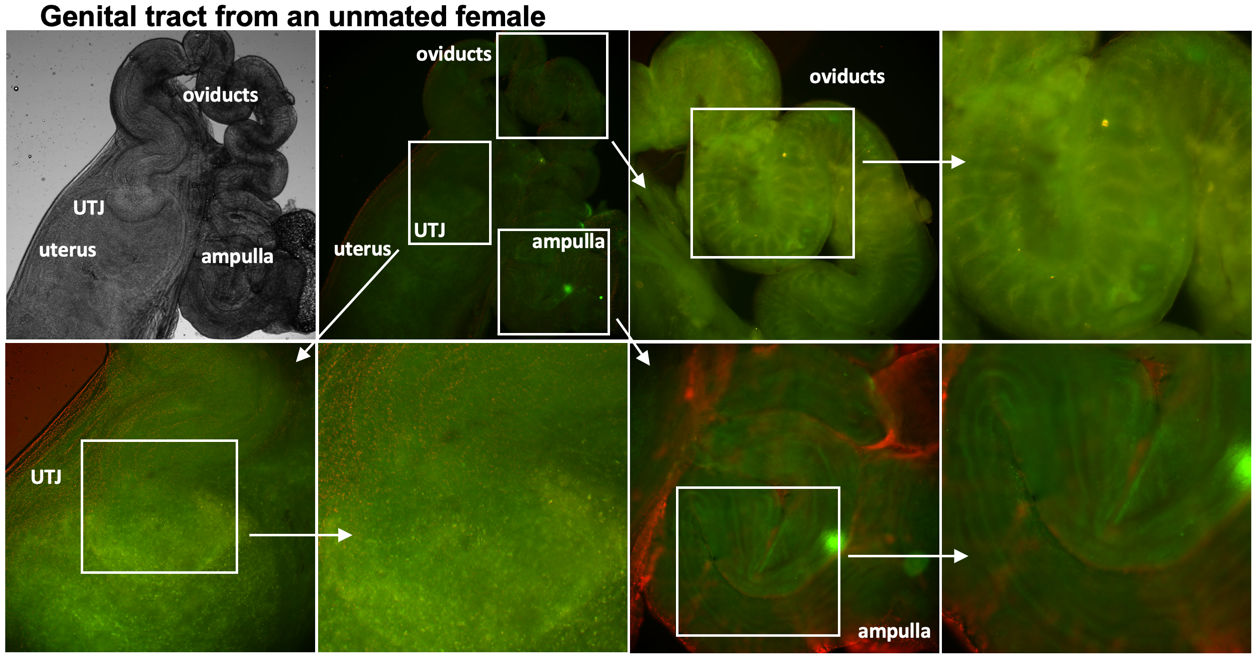


**Figure S5.** Female genital tract from an unmated female, depicting the tissue´s intrinsic autofluorescence.

**Supplemental Movie 1.** Motility of epididymal WT and sAC^-/-^ sperm incubated in the absence (-) or presence of 20 mM HCO_3_^-^ and 5 mg/ml BSA (+HCO_3_^-^/BSA) or 0.5 mM 8Br-cAMP and 0.5 μM IBMX (+cAMP) for 20 min. Shown are representative movies with tracks generated by CASA (IVOSII Hamilton Thorne). Track color code: motile (green), progressive (turquoise), hyperactivated (pink), static (red), not counted (yellow).

**Supplemental Movie 2.** Motility of ejaculated WT and sAC^-/-^ sperm incubated in the absence (-) or presence of 20 mM HCO_3_^-^ and 5 mg/ml BSA (+HCO_3_^-^/BSA) or 0.5 mM 8Br-cAMP and 0.5 μM IBMX (+cAMP) for 20 min. Shown are representative movies with tracks generated by CASA (IVOSII Hamilton Thorne). Track color code: motile (green), progressive (turquoise), hyperactivated (pink), static (red), not counted (yellow).
